# Anionic Lipid Catalyzes the Generation of Cytotoxic Insulin Oligomers

**DOI:** 10.3390/biom15070994

**Published:** 2025-07-11

**Authors:** Jhinuk Saha, Audrey Wolszczak, Navneet Kaur, Malitha C. Dickwella Widanage, Samuel D. McCalpin, Riqiang Fu, Jamel Ali, Ayyalusamy Ramamoorthy

**Affiliations:** 1 National High Magnetic Field Laboratory, Florida State University, 1800 E. Paul Dirac Drive, Tallahassee, FL 32310, USA; aaw21a@fsu.edu (A.W.); nk22u@fsu.edu (N.K.); malitha.dickwella@nrel.gov (M.C.D.W.); sdm24f@fsu.edu (S.D.M.); rfu@magnet.fsu.edu (R.F.); jali@eng.famu.fsu.edu (J.A.); 2 Department of Chemical and Biomedical Engineering, FAMU–FSU College of Engineering, Florida State University, 2525 Pottsdamer St., Tallahassee, FL 32310, USA; 3 Institute of Molecular Biophysics, Florida State University, 91 Chieftan Way, Tallahassee, FL 32304, USA

**Keywords:** insulin, amyloids, aggregation, lipids

## Abstract

The misfolding and aggregation of proteins into amyloidogenic assemblies are key features of several metabolic and neurodegenerative diseases. Human insulin has long been known to form amyloid fibrils under various conditions, which affects its bioavailability and function. Clinically, insulin aggregation at recurrent injection sites poses a challenge for diabetic patients who rely on insulin therapy. Furthermore, decreased responsiveness to insulin in type 2 diabetic (T2D) patients may lead to its overproduction and accumulation as aggregates. Earlier reports have reported that various factors such as pH, temperature, agitation, and the presence of lipids or other proteins influence insulin aggregation. Our present study aims to elucidate the effects of non–micellar anionic DMPG (1,2–dimyristoyl–sn–glycero–3–phosphoglycerol) lipids on insulin aggregation. Distinct pathways of insulin aggregation and intermediate formation were observed in the presence of DMPG using a ThT fluorescence assay. The formation of soluble intermediates alongside large insulin fibrils was observed in insulin incubated with DMPG via TEM, DLS, and NMR as opposed to insulin aggregates generated without lipids. ^13^C magic angle spinning solid–state NMR and FTIR experiments indicated that lipids do not alter the conformation of insulin fibrils but do alter the time scale of motion of aromatic and aliphatic side chains. Furthermore, the soluble intermediates were found to be more cytotoxic than fibrils generated with or without lipids. Overall, our study elucidates the importance of anionic lipids in dictating the pathways and intermediates associated with insulin aggregation. These findings could be useful in determining various approaches to avoid toxicity and enhance the effectiveness of insulin in therapeutic applications.

## 1. Introduction

Amyloid aggregation has evolved as a key underlying mechanism of several human diseases. For instance, diseases like type 2 diabetes (T2D), Parkinson’s disease (PD), Alzheimer’s disease (AD), Huntington’s disease, and amyotrophic lateral sclerosis (ALS) involve the deposition of amyloid aggregates of one or more proteins within their pathophysiology [1,2,3,4,5]. Amyloid aggregation and insulin ball formation under the skin has also been observed in diabetes patients who regularly take insulin injections. Several patients have been observed to develop tissue necrosis and severe infection around insulin injection sites due to insulin amyloids [6,7,8,9]. The presence of insulin aggregates in injection sites as well as in the bloodstream therefore poses a challenge for insulin to function effectively as a therapeutic [8,10]. Insulin is a peptide hormone mainly involved in glucose homeostasis by cells as well as cellular growth and metabolism. It is a 51–amino acid protein that contains peptide chains A (30 amino acids) and B (21 amino acids) connected with 3 disulfide linkages [11,12]. Most insulin production in the human body occurs in β cells of the pancreas and is controlled by glucose levels in the blood [13,14,15]. Insulin concentration in the β cells of the pancreas, where it is mainly produced and stored, has been estimated to be about 100 mM [16]. Once generated, insulin is stored within secretory granules as zinc–bound hexamers at concentrations of roughly 40 mM [17,18]. However, to become functional, insulin hexamers dissociate to monomers upon release from β cells [18]. The amount of insulin secreted per day in a non–type 2 diabetic individual is around 45.5 U [19], which is equivalent to about 270 µM per day [20]. In patients with type 2 diabetes, about 20 U of insulin is typically injected per day, which is equivalent to about 120 µM [21]. Depending on the local environment, such as the presence of different pH or temperature, insulin monomers can undergo partial unfolding and aggregate into amyloid species characterized by peptide strands arranged in repetitive cross β–sheet structures stabilized by hydrogen bonds [22,23,24,25,26]. Insulin aggregation is dependent on concentration, pH, T, and mechanical agitation. For example, an increase from 30 µM to 3.2 mM bovine insulin concentration dramatically shortens the lag time from 4 to ~0 h under pH 1.6 and 60 °C without agitation, whereas the lag time is reduced to ~1 h at 37 °C and pH 1.6 under vigorous agitation [27]. These amyloid aggregates are known to be inactive for cellular uptake and metabolism and have been reported to result in cellular toxicity [28,29,30].

Previously, it has been reported that the conformation and structural arrangements of monomeric units within insulin fibrils contain 2, 4, and 6 protofilaments [31]. Additionally, Reif and coworkers have reported that in insulin fibrils generated under low pH, insulin monomers adopt a U–shaped conformation and fold into four β strands in two layers within each fiber unit [32]. Amyloid aggregates of various disease–related amyloid proteins are widely known to exhibit structural– and molecular–level polymorphism [33,34]. Furthermore, several proteins with well–defined folded structures have also been reported to form polymorphic fibrillar structures [35]. Importantly, correlations between such polymorphic fibril or oligomeric structures have been established with their stability and pathological outcomes [36,37]. Similarly, other studies have shown that polymorphic fibrillar aggregates possess prion–like properties [38]. Although different morphologies of fibrillar aggregates of insulin have been reported previously [39,40,41], the molecular details of insulin aggregation in the presence of lipids remain unclear.

Polymorphism within fibril structures can arise from several different phenomena: (1) environmental factors such as pH, temperature, agitation, ionic strength, etc. [42,43,44,45,46,47,48,49] or (2) interactions of the peptides with cofactors such as other proteins, lipids, metal ions, sugars, etc. [50,51]. Such factors can modulate the pathways of amyloid aggregation, generating kinetically trapped intermediates or ultimate aggregates that vary in toxicity [52,53,54]. Several reports have previously shown that different polymorphs of insulin—such as fibrils, hexamers, or spherulites [55,56]—can form under various environmental conditions. For example, insulin polymorphs can arise due to low pH, the presence of D_2_O, elevated temperatures, changes in ionic strength, or the presence of surfactants and lipids [22,23,27,57]. Negatively charged lipid vesicles have been reported to promote and zwitterionic lipids have been shown to inhibit insulin aggregation. These aggregates, mainly the oligomeric intermediates, have also been shown to cause cellular damage through distinct mechanisms [58,59]. Since insulin is a peptide hormone that regulates glucose uptake by the cells in different parts of the body, it is susceptible to exposure to variable pH and ionic strength conditions to form amyloid structures, such as acidic conditions in the secretory granules and neutral conditions in the cytoplasm and extracellular matrix [60]. Additionally, insulin is also prone to interact with free lipids, which are abundant in plasma and blood as cleavage products of triglycerides [61]. Therefore, it is important to investigate the amyloidogenic interaction of insulin with such lipids below their critical micelle concentration.

In this study, we report our investigation on the effects of free lipids (i.e., below critical micellar concentration (CMC)) on insulin aggregation. Results obtained from the effects of two different lipids that differ in charge and CMC on 80 μM insulin are reported: anionic DMPG (1,2–Dimyristoyl–sn–glycero–3–phosphoglycerol) lipid, with a CMC of 11 μM, and zwitterionic DMPC (1,2–Dimyristoyl–sn–glycero–3–phosphocholine) lipid, with CMC of 6 nM. These protein–to–lipid ratios were chosen based on preliminary optimization to ensure specific, sub–CMC interactions without inducing micelle formation or nonspecific aggregation. This design allowed us to observe lipid–dependent effects at physiologically relevant and mechanistically informative concentrations and aligns with previous studies that emphasize the importance of sub–CMC lipid–protein interactions. Based on thioflavin–T (ThT) fluorescence assay, we found that the aggregation kinetics of insulin were altered by the presence of an anionic phospholipid, 1,2–Dimyristoyl–sn–glycero–3–phosphoglycerol (DMPG), below its critical micelle concentration (CMC). Our results show that DMPG triggers the generation of low–molecular–weight cytotoxic oligomers alongside longer fibrils in contrast to the nontoxic insulin fibrils formed under low pH. CD, FTIR, and solid–state NMR experiments show that the insulin fibrils formed along with β–sheet oligomers in the presence and absence of anionic phospholipids are β–sheet structured with a rigid core. Solid–state NMR spectra reveal a similar conformation for the fibrils grown in the presence and absence of anionic and zwitterionic lipids. Importantly, the DMPG–induced insulin oligomers exhibit high cellular toxicity.

## 2. Experimental Section

### 2.1. Materials

Recombinant human insulin was obtained from Roche (Indianapolis, IN, USA). Dimyristoylphosphatidylglycerol (DMPG) and dimyristoylphosphatidylcholine (DMPC) lipids were purchased from Avanti Polar Lipids (Alabaster, AL, USA). Sodium chloride (NaCl), sodium phosphate, and trypsin–EDTA were purchased from Fisher Scientific (Hampton, NH, USA). Dulbecco’s Modified Eagle Medium (DMEM) was procured from Gibco (Grand Island, NY, USA). Copper grids used for transmission electron microscopy were supplied by Millipore Sigma (Burlington, MA, USA), and Uranyless Stain was purchased from Electron Microscopy Sciences. Thioflavin T (ThT) was obtained from Millipore Sigma (Burlington, MA, USA).

### 2.2. Insulin Monomer Preparation and Thioflavin–T–Based Fluorescence Experiments

Insulin solution was prepared by dissolving the desired amount of insulin in 10 mM sodium phosphate buffer at pH 3.0 and briefly vortexed to obtain a clear solution, and the concentration was measured using a UV–vis spectrophotometer (Implen, Westlake Village, CA, USA) [62]. Subsequently, ThT fluorescence kinetics assays to study the self–assembly and aggregation of insulin in the presence and absence of different concentrations of DMPG were carried out on a Biotek Synergy H1 instrument (Agilent Technologies, Winooski, VT, USA) with excitation at 452 nm and emission at 485 nm. Briefly, 80 µM insulin was incubated with 2, 4, 6, or 8 µM DMPG lipids or without any lipids in 10 mM sodium phosphate buffer of pH 3 with 50 µM ThT and 150 mM NaCl at 37 °C. Fluorescence was read in 96–well plates for 24–48 h under orbital shaking at 700 rpm and with an interval of 15 min. Data shown was repeated at least three times independently to ensure reproducibility. Aggregation lag times from ThT fluorescence traces were estimated by fitting the data to a sigmoidal curve using the Boltzmann equation in OriginLab8.

### 2.3. TEM Experiments

Samples were freshly prepared by applying 10 µL of reaction samples to 300 mesh–size Formvar/Carbon Supported copper grids (Millipore Sigma, Burlington, MA, USA, catalog #TEM–FCF300CU) and dried for 10 min. Subsequently, the grids were stained with 10 µL of Uranyless stain (EMS, Hatfield, PA, USA, catalog #22409); any excess stain was soaked on a blotting paper, and the grid was dried at room temperature for 10 more minutes. Samples were imaged using an HT7800 Hitachi TEM microscope (Minato-ku, Tokyo, Japan) at an acceleration voltage of 100 kV. Images were collected from at least three grid regions for independent samples at magnifications between 15,000× and 25,000×.

### 2.4. CD Experiments

Insulin reaction samples and the resulting fibril samples were diluted accordingly to obtain a final concentration of 20 µM, and the supernatant samples were kept undiluted; 10 mM sodium phosphate buffer at pH 7.4 was used as a blank. CD spectra were obtained between 200 and 260 nm in a Chirascan instrument (Leatherhead, Surrey, UK) at room temperature. An average of three scans for each sample were plotted using OriginPro software 2024. The CD spectra were analyzed using BestSel software (v1.3.230210) to estimate the secondary structure of insulin [63].

### 2.5. DLS Experiments

Dynamic Light Scattering (DLS) measurements were performed using a NANOSTAR DLS instrument (Wyatt Technology Corporation, Santa Barbara, CA, USA) to determine the hydrodynamic size distribution of insulin aggregates. For each measurement, a 5 µL sample of 5–10 µM concentration was loaded into the quartz cuvette. The measurements were conducted at room temperature (RT) under standard operating conditions of the instrument. Each sample was equilibrated for 2 min before measurement. Data was collected from at least three independent measurements and analyzed using the instrument’s proprietary software.

### 2.6. FTIR Experiments

FTIR spectra were acquired using a Thermo Nicolet with diamond–ATR accessory (Waltham, MA, USA), and 1 mg samples of lyophilized insulin (insulin fibrils, oligomers, and monomers) were scanned from 1500 to 1800 cm^−1^ at a resolution of 8 cm^−1^. A total accumulation of 512 spectral scans was obtained per sample, and the data were baseline corrected, deconvoluted, and plotted using OriginLab8.

### 2.7. Solid–State NMR Experiments

Solid–state NMR experiments were performed using Bruker Avance spectrometers operating at 600 MHz (14.1 T) and 850 MHz (20.0 T) at the National High Magnetic Field Laboratory (NHMFL), Tallahassee, FL, USA, and at 600 MHz (14.1 T) at Bruker facilities (Fällanden, Switzerland). The insulin fibrils (~1 mg) were packed into a 3.2 mm rotor for experiments using an HCN triple–resonance MAS probe (NHMFL, home–built), an HX double–resonance MAS probe (NHMFL, home–built), and an HCN triple–resonance CryoProbe (Bruker). ^13^C CP–MAS and refocused–INEPT spectra were recorded under MAS spinning speeds of 12–12.5 kHz at various temperatures. Typical radiofrequency field strengths used were 68–83 kHz for ^1^H decoupling, 62.5 kHz for ^1^H hard pulses, and 50–62.5 kHz for ^13^C pulses. Experimental parameters such as cross–polarization contact time (τ_HC_), sample temperature (T), recycle delay (d1), number of scans (NS), and *J*–evolution times are provided in the figure legend. ^13^C chemical shifts were externally referenced to adamantane’s CH_2_ peak at 38.48 ppm with 0 ppm for ^13^C peak of tetramethylsilane (TMS).

### 2.8. Solution–State NMR Experiments

Solution NMR experiments were performed on a 700 MHz Bruker NMR spectrometer equipped with a 5 mm TCI cryoprobe. NMR samples were prepared and kept on ice before being loaded in the NMR probe for data acquisition. ^1^H NMR data was acquired with 1024 scans, 1.25 s recycle delay, and water suppression by the WATERGATE sequence. Spectra were collected at 35 °C, and the sample was kept in the magnet at the same temperature between data acquisitions at different timepoints.

### 2.9. Cellular Toxicity

NIH3T3 fibroblast cells were used for cytotoxicity assays to evaluate the general cellular toxicity of the amyloid aggregates in a non–neuronal context. Fibroblasts like NIH3T3 are well characterized, robust, and commonly used for baseline toxicity assessments due to their reproducibility and ease of culture. This provides a foundational understanding of whether the observed cytotoxic effects stem from broad membrane–disruptive or oxidative mechanisms rather than being exclusive to neuronal pathways. NIH3T3 fibroblasts were maintained in Dulbecco’s Modified Eagle Medium (DMEM) supplemented with 10% fetal bovine serum (FBS) and 1% penicillin–streptomycin. The cells were maintained at 37 °C in a humidified incubator with 5% CO_2_. For subculturing, when the cells reached 70–80% confluency, the culture medium was removed, and the cells were washed with phosphate–buffered saline (PBS). Then, 0.25% trypsin–EDTA solution was added to detach the cells, which were centrifuged to obtain a pellet. This pellet was resuspended in fresh culture medium and transferred to new culture vessels to ensure optimal growth conditions for experimental purposes. NIH3T3 cells (1 × 10^4^) were seeded in 96–well plates and treated with different aggregate species of insulin for 36 h. After treatment, cellular viability was assessed using the CCK–8 assay, and absorbance was measured at 450 nm to evaluate cell viability. The microscope used was an Invitrogen™ EVOS™ M7000 Imaging System (Carlsbad, CA, USA) with a 40× objective. 

## 3. Results and Discussion

### 3.1. Anionic Phospholipids Below Their Critical Micelle Concentration Augment Insulin Aggregation

**Figure 1:**
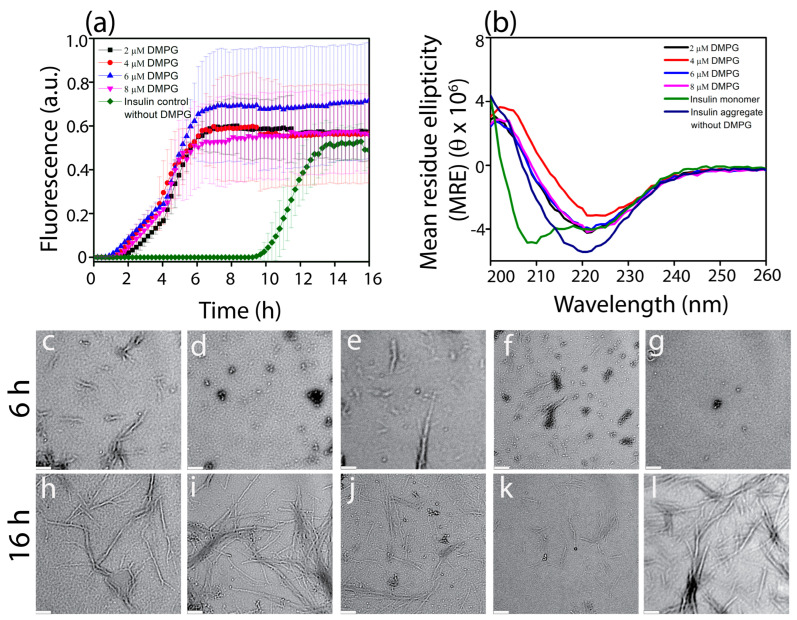
ThT fluorescence kinetics (**a**) and circular dichroism (CD) spectra (**b**) of 80 µM insulin with 0 (◆), 2 (■), 4 (●), 6 (▲), and 8 µM (▼) DMPG. Insulin and DMPG were incubated in 10 mM sodium phosphate buffer (pH 3, 150 mM NaCl, 50 µM ThT) at 37 °C and with agitation at 700 rpm, while insulin monomers were freshly prepared in 10 mM sodium phosphate buffer at pH 3. (**c**–**l**) TEM images of insulin aggregates generated in the ThT fluorescence assay with 2 (**c**,**h**), 4 (**d**,**i**), 6 (**e**,**j**), and 8 (**f**,**k**) µM DMPG and without DMPG (**g**,**l**); these aggregates were collected after 6 h (**c**–**g**) or 16 h (**h**–**l**). The indicated scale bar is 200 nm.

To investigate the effect of negatively charged phospholipids below their critical micelle concentration on amyloid formation by insulin monomers, we performed ThT fluorescence assays of 80 µM insulin incubated with 2–8 µM DMPG phospholipids, which is below the reported CMC of DMPG (i.e., 11 µM [64]) (Figure 1a). We found that the presence of DMPG accelerated insulin aggregation drastically, shortening the lag time to 1–2.5 h after the start of the reactions, whereas the insulin aggregation exhibited a lag time up to 10 h in the absence of lipids (Figure S1a–c). While these results support previous findings on the acceleration of insulin aggregation in the presence of anionic lipids [65], we found that, within the lipid concentration range used here, the ThT fluorescence kinetics were independent of the concentration of lipids in the sample. To further investigate the underlying molecular mechanism of the aggregation pathway, we used Amylofit [66], an amyloid fitter software, with its pre–existing protein aggregation models. Since the global fitting of insulin aggregation at different concentrations has already been reported using AmyloFit [67,68], we used AmyloFit to analyze our data obtained from 80 µM insulin (Figure S2b,d). The AmyloFit of our data indicated that insulin aggregation kinetics without lipids followed a secondary nucleation pathway, which is in agreement with previously reported data [67,68]. On the other hand, AmyloFit of data obtained from insulin monomers in the presence of DMPG exhibited a secondary nucleation pathway with saturating elongation (unseeded) (Figure S2a,c). Although the secondary nucleation pathway with saturating elongation (unseeded) model fits the data well for the DMPG–insulin samples, it is important to note that the model does not account for the DMPG lipid concentration in the sample, which may create a catalytic interface and influence the formation of the primary nucleus and the overall aggregation mechanism, as noted previously with other studies [69]. We also attempted to fit our data using other models, such as fragmentation (Figure S2e,h) and primary nucleation (Figure S2f,g), for insulin with and without DMPG lipids. But these models did not provide a good fit to our data (see Supplementary Figure S2). Additionally, we screened the effect of zwitterionic DMPC (1,2–Dimyristoyl–sn–glycero–3–phosphocholine) lipids across various concentrations near its CMC (6 nM) [64,70,71]. We found that DMPC has a negligible effect on insulin at and below the CMC; however, it delays insulin aggregation up to 4 h above CMC at 8 nM (Figure S3). We further performed a secondary structure analysis of the bulk solution at 16 h after the start of the aggregation reaction with CD spectroscopy. We observed that by 16 h, the alpha helical structure of insulin at *t* = 0 h, as indicated by the double minima at 222 and 210 nm, had transitioned to a β–sheet structure, which was indicated with a minimum at 218 nm [72].

Previous work has demonstrated that amyloid aggregation and the formation of fibrils are preceded by the generation of intermediate oligomeric structures [53,73,74]. The structure, conformation, and stability of such oligomers are dependent upon environmental factors such as temperature, pH, ionic strength of the buffer, or the presence of other interacting molecules such as proteins and lipids [22,27]. Therefore, to investigate whether the presence of DMPG lipids affects the generation of intermediate species along the aggregation pathway of insulin, we performed transmission electron microscopy (TEM) imaging on insulin aggregates generated with various concentrations of DMPG (Figure 1c–l). Samples were collected after incubation at 37 °C and under continuous shaking at 700 rpm for 6 and 16 h. We observed that with 2 µM DMPG, insulin formed short fibrils within 6 h of incubation (Figure 1c) that became larger and denser by 16 h (Figure 1h). However, when the concentration of DMPG was increased to 4, 6, and 8 µM, a mixture of small spherical oligomeric intermediates and short fibrils of insulin emerged within 6 h of incubation (Figure 1d–f). While the intermediates largely transitioned to larger fibrils by 16 h in the case of 4 µM DMPG (Figure 1i), the oligomeric intermediates persisted alongside larger fibrils after 16 h for insulin samples with 6 µM and 8 µM DMPG (Figure 1j,k). Insulin aggregated without lipids or with DMPC lipids resulted in the formation of a small population of spherical intermediates at 6 h (Figure S3b–f and Figure 1g) that completely turned into larger fibrils within 16 h (Figure S3g–k and Figure 1l). Therefore, our findings suggest that insulin aggregates via an alternative pathway by forming distinct oligomeric species in the presence of certain concentrations of anionic phospholipids. We also attempted to investigate the formation of different intermediate species upon insulin aggregation with solution NMR as performed previously in other studies related to amyloid aggregation [75,76]. We observed similar peaks in ^1^H spectra at 0 h and at 24 h for insulin samples incubated at 35 °C with or without DMPG but without shaking the sample in the NMR tube (Figure S4). The absence of peak broadening or intensity loss is indicative of negligible or delayed aggregation [77]. These observations emphasize the significance of agitation for insulin aggregation.

### 3.2. Small Soluble Cytotoxic Oligomers Are Generated Alongside Larger Fibrils

**Figure 2:**
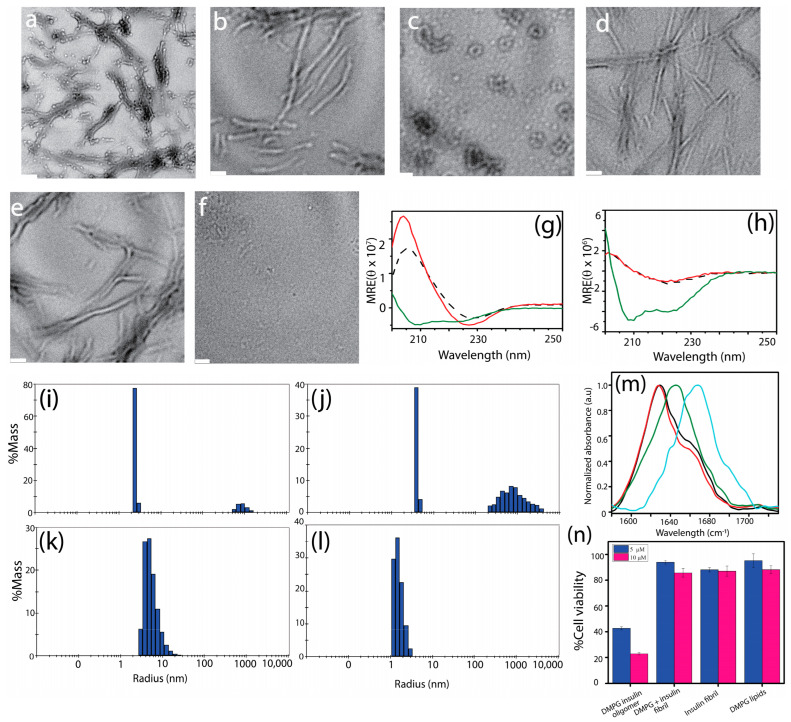
TEM images of total (**a**,**d**), pellet (**b**,**e**), and supernatant (**c**,**f**) fractions of 80 µM insulin monomer reaction with (**a**–**c**) and without (**d**–**f**) 6 µM DMPG. The scale bar is 100 nm. DLS profiles (**i**–**l**) and CD spectra (**g**,**h**) of pellet (**i**,**j**) and supernatant (**k**,**l**) fractions of 80 µM insulin monomer reaction with ((**i**,**k**) in DLS; black trace in CD) and without ((**j**,**l**) in DLS; red trace in CD) 6 µM DMPG centrifuged at 18,000× *g* for 30 min after incubation at 70 °C under continuous agitation at 700 rpm for 16 h. CD spectra of insulin monomers at 0 h (green traces in (**g**,**h**)). (**m**) FTIR spectra of insulin fibrils generated with (ꟷ) and without (ꟷ) DMPG lipids, insulin oligomers generated with DMPG lipids (ꟷ), and insulin monomers (ꟷ). (**n**) CCK–8 cytotoxicity assay on NIH3T3 cells for 5 and 10 µM insulin oligomers catalyzed by DMPG and insulin fibrils generated with and without DMPG.

TEM images of insulin aggregates generated in presence of 6 µM and 8 µM DMPG indicate that a mixture of small oligomers and fibrils was present in the reaction sample and stable for at least 16 h (Figure 1j,k). When the temperature was increased to speed up the aggregation process, we observed that the oligomers were still generated alongside fibrils. For example, incubation of 6 µM DMPG and insulin as previously, except at a higher temperature (70 °C), resulted in a mixture of oligomers and fibrils in the presence of DMPG (Figure 2a) but not in its absence (Figure 2d). Next, we isolated the insoluble fibril fraction from the soluble oligomeric fraction using high–speed centrifugation. The samples containing the insoluble fibril pellet formed with or without DMPG displayed a large hydrodynamic diameter greater than 1000 nm (Figure 2i,j), as observed by DLS. Another size distribution peak was also observed near the smaller diameter region at 1–10 nm, which may be due to dissociated monomeric or oligomeric species. It should also be noted that the DLS approximates particles as spheres and essentially provides the hydrodynamic radii of the particle rather than the physical dimension of the fibrils. The approximate hydrodynamic radius of the particle depends on the fibril orientation as well as the fibril morphology. The presence of large fibrils in these samples was further confirmed by TEM (Figure 2b,e and Figure S5a–c,m–o). DLS profiles of the soluble fractions revealed the presence of oligomeric structures of about 10–15 nm for insulin incubated with DMPG (Figure 2k), whereas species with diameters of 2–4 nm corresponding to insulin monomers (Figure S6) were observed in the soluble fraction of insulin incubated without DMPG (Figure 2l). Accordingly, small spherical structures were visible under TEM for the soluble fraction of insulin incubated with DMPG (Figure 2c and Supplemental Figure S5d–f). TEM images of 6 µM DMPG show no micelle–like structures (Figure S7a–d), confirming that the oligomeric structures seen in the insulin–DMPG samples are not from DMPG micelles. In contrast, negligible aggregate species were visible in the soluble fraction of insulin aggregates formed without lipids (Figure 2f and Supplemental Figure S5p–r). The secondary structure of both pellet and supernatant for each sample was further analyzed using CD spectrometry. It was observed that all fibril samples showed the presence of twisted β–sheet structure evident from a minimum near 230 nm [78] as opposed to insulin monomers, which mostly had α–helical structures evident from the double minima near 210 and 222 nm (Figure 2g,h). Furthermore, we analyzed the CD spectra using Bestsel [63] to estimate the secondary structure of insulin monomers, DMPG–insulin fibril pellets, DMPG–insulin oligomeric supernatant, and insulin fibril pellets and its supernatant. CD spectral analysis revealed ~100% α–helical structure for insulin monomers and ~50% of distorted helical and ~50% β–turn for fibril pellets generated in the presence or absence of DMPG lipids (Table S1). On the other hand, the supernatant of the DMPG–insulin sample showed the presence of a mixture of parallel and antiparallel β–sheets; and the supernatant of the insulin aggregates without lipids showed a distorted helical structure, indicating unreacted or dissociated monomers (Table S1). Additionally, the presence of peaks at 1638 cm^−1^ and 1699 cm^−1^ in the oligomeric fraction of the DMPG and insulin sample suggests the presence of both parallel and antiparallel β–sheets, which agrees with our CD data (Figure S8a). The presence of β–sheets in insulin fibrils both with and without DMPG was confirmed by the peaks near 1626 cm^−1^ in the FTIR spectra (Figure 2m and Figure S8b,c). The presence of β–turns was also confirmed by the peaks at 1662 and 1675 cm^−1^ for insulin fibrils generated under DMPG, whereas the fibrils generated without lipids indicated a β–turn peak around 1666 cm^−1^ (Figure S8b,c). The oligomeric fraction of DMPG and insulin displayed a shifted peak at 1668 cm^−1^, which is attributed to β–turns [79,80]. In contrast, the FTIR spectrum of insulin monomers displayed a peak at 1644 cm^−1^, indicating a mixture of α–helical and disordered conformations (Figure S8d). For additional quantitative information, neither DLS nor NMR measurements proved to be effective in providing a precise quantification of the insulin aggregates. Insulin oligomers are too large for detection by solution NMR (Figure S9). While small oligomers are detectable by DLS, very large fibrils cannot be accurately quantified due to more intense scattering of light.

**Figure 3:**
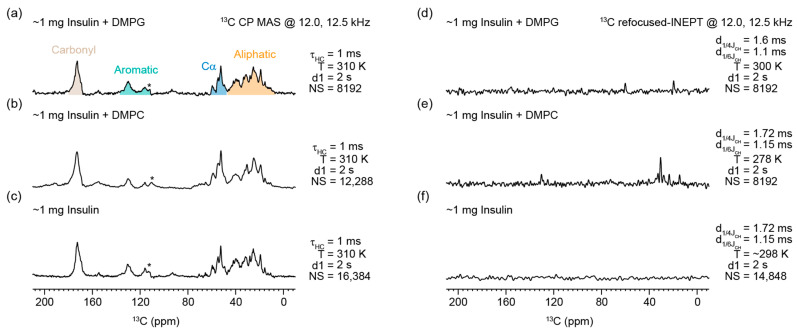
Natural abundance ^13^C MAS NMR spectra of insulin aggregates prepared in the presence of DMPG and DMPC (**a**,**b**,**d**,**e**) and without lipids (**c**,**f**). The CP–MAS spectra were obtained at 12 kHz (**a**,**c**,**d**) and at 12.5 kHz (**b**,**e**,**f**) at the indicated contact time (τ_HC_) for cross–polarization, temperature (T), recycle delay (d1), and number of scans (NS). ^13^C MAS NMR spectra obtained using refocused–INEPT showed no peaks from insulin fibers generated without lipids (**f**), whereas a few peaks were observed for the insulin–lipid samples (**d**,**e**), which are likely to be from aromatic and aliphatic side chains of insulin, as the samples had very small amounts of lipids. All spectra were obtained on a 600 MHz Bruker NMR spectrometer except the refocused–INEPT spectra, which were acquired using an 850 MHz Bruker NMR spectrometer. Additional CPMAS NMR spectra are given in Figure S10. * is the spinning side band.

To elucidate the structural features of insulin fibrils, magic angle spinning (MAS) solid–state NMR experiments were conducted on the fibrils. The natural abundance ^13^C CP–MAS NMR spectra of insulin fibrils, prepared in the absence and presence of DMPG and DMPC lipids, were found to be similar (Figure 3a–c and Figure S10a–f). These results indicated that the backbone conformation of insulin fibrils was not altered by the presence of lipids (DMPG or DMPC), as evidenced by the similar ^13^Cα and ^13^CO chemical shifts observed for all three samples. These findings are in good agreement with the observations from FTIR spectroscopy. To investigate the side–chain environments of the insulin fibrils, refocused–INEPT experiments were utilized. The observed peaks in refocused–INEPT spectra were predominantly attributed to fibril side chains, since the fibrils generated under free lipids (6 μM DMPG or 6 nM DMPC) were expected to contain negligible lipids due to discarding the lipid–containing soluble fraction via centrifugation prior to NMR measurements. A comparison of the refocused–INEPT spectra revealed notable differences between the fibrils formed in the presence of DMPG and DMPC (Figure 3d–f). Specifically, the DMPC–catalyzed fibrils exhibited distinct aliphatic and aromatic signals (Figure 3d,e), suggesting subtle structural variations in the fibrils influenced by the lipid environment. These findings suggest that the fibrils formed in the presence of DMPG and DMPC are likely to exhibit distinct structural arrangements. These observations indicate that the lipid environment influences the fibril structure and potentially alters the conformational flexibility or intermolecular interactions. This variation underscores the impact of lipid–based interactions on the kinetics of fibril formation and structure of insulin aggregates. In order to probe the interaction between monomeric insulin and lipids, ^1^H NMR spectra of monomers of insulin in the presence of DMPG were acquired for 24 h (Figure S4). However, no measurable changes in the spectrum were observed, indicating no aggregation of insulin monomers. This further confirms the need for mechanical agitation to induce the aggregation of insulin monomers. On the other hand, solution NMR spectra (Figure S9) of insulin samples at different timepoints of aggregation under agitation with or without lipids were found to be significantly different from those of insulin monomers (Figure S4). These results suggest that insulin quickly forms aggregates (at least within 6 h) that are large in size for detection by solution NMR and, therefore, do not provide narrow ^1^H spectral lines in solution NMR spectra. The observed peaks in ^1^H NMR spectra of the agitated insulin samples (with or without lipids) are likely to be from monomers present in the samples. Additional solid–state and solution NMR experiments using isotopically (^13^C) enriched insulin samples could be useful to obtain piercing insights into lipid–insulin interactions.

Previous research by several groups has established that amyloid aggregates of the same protein generated under different conditions display variable toxicities towards mammalian cells. Therefore, we analyzed the cytotoxic effects of different aggregate species of insulin towards NIH3T3 fibroblast cells. Fibril pellets generated with and without DMPG lipids and oligomers generated with DMPG lipids were incubated with NIH3T3 cells for 36 h. Then, a CCK–8 assay was carried out to assess the dehydrogenase inactivation in cells as a measure of cellular viability. Low toxicity was observed in the case of fibril pellets of insulin samples with and without DMPG in a dose–dependent manner (Figure S11a). Interestingly, the soluble fraction from insulin–DMPG samples containing the insulin oligomers showed the highest toxicity where cellular viability decreased to 40% and 20% with 5 and 10 µM insulin concentrations, respectively (Figure 2n). Brightfield microscopy images of distorted cellular morphology upon the addition of insulin oligomers or fibrils compared with healthy control cells can be seen in Figures S12 and S13. To observe the dose–dependent response of insulin oligomers on NIH3T3 cells, CCK–8 assay was performed for 2.5, 5, 10, and 15 µM insulin oligomers generated in the presence of DMPG lipids and showed a decrease in cell viability with increased concentration of oligomer dosage (Figure S11b).

### 3.3. Discussion

Our study underscores the impact of the anionic phospholipid DMPG on insulin aggregation, showing distinct aggregation pathways that differ from those observed under low pH conditions without lipids. While low pH may not directly be physiologically relevant, we used it in this study to enhance the solubility of insulin (pI~5.3) like in previously reported studies [27,58,59,65,81]. The presence of negatively charged phospholipids has been shown to accelerate the aggregation of insulin in our study as well as in previous studies [62]. These findings suggest that the negative charge of lipids plays a crucial role in lipid–protein interactions and in the self–assembly process leading to protein aggregation. The interaction between the negatively charged lipids and insulin can be explained by electrostatic interactions. Since insulin has a pI of 5.3, the net charge on the insulin in acidic pH is positive. Therefore, electrostatic interaction between the positively charged insulin (Figure S14a) and negatively charged DMPG (Figure S14b) is likely to facilitate insulin–insulin interactions, promoting the self–assembly process to form the oligomers and fibrils independent of the concentration of DMPG lipids used. It is important to note that DMPG may not necessarily act in a simple, concentration–dependent manner, as its role in reshaping insulin aggregation could be more complex. For instance, DMPG, even at below critical micelle concentration, could influence aggregation by stabilizing certain intermediates rather than merely altering the overall aggregation rate. This mechanism may differ significantly from insulin aggregation in the absence of lipids even under similar conditions, resulting in a much longer lag time of 10 h, as shown in Figure 1a. Additionally, other factors, such as agitation or temperature, may also contribute to the observed effects. On the other hand, minimal interaction was observed when insulin was incubated with DMPC below its CMC (Figure 1 and Figure S3). The zwitterionic DMPC lipids are likely to interact with the positively charged insulin through weaker hydrophobic interactions. These interactions may stabilize partially unfolded insulin in a way that permits only limited nucleation and elongation of amyloid fibrils, resulting in delayed aggregation and the formation of shorter fibrils (Figure S3). Insulin aggregation was somewhat attenuated in the presence of DMPC above its CMC. Interestingly, it is also noted that a change in the rate of aggregation corresponded with changes in the secondary structure of insulin. While incubation of insulin with DMPG led to the formation of small spherical oligomers and larger fibrils, incubation with DMPC resulted in a mixture of shorter and larger fibrillar structures (Figure 2a–c and Figure S5g–l). Previous studies have emphasized the critical role that membranes and membrane components play in facilitating the aggregation of various amyloidogenic proteins [82]. These proteins have demonstrated a tendency to interact with lipid environments, resulting in altered aggregation pathways. Key lipid properties such as chain length, head–group charge, and overall lipid composition are known to significantly influence these interactions, leading to distinct aggregation behaviors that may produce structurally and functionally unique assemblies with varying toxicities [83,84,85]. Our study observed that the presence of DMPG (a negatively charged lipid) below its critical micelle concentration promoted the formation of oligomeric clusters, a behavior reminiscent of the cluster formation seen in α–Synuclein with increasing negative charge density [86]. This shared mechanism underscores the importance of lipid–protein interactions in modulating aggregation dynamics. Furthermore, our findings suggest that distinct intermediate species are formed during the early stages of aggregation in lipid–rich environments, ultimately leading to the development of larger fibrillar assemblies. These results highlight a potentially universal mechanism in which anionic lipids serve as key modulators of amyloid aggregation by stabilizing intermediate species, which may vary in structure and toxicity depending on the specific lipid–protein interaction.

Based on the experimental results reported in this study, we propose that under certain concentrations of DMPG, insulin aggregates through a different aggregation pathway due to the presence of oligomeric aggregates. It is possible that the decrease in the lag time also correlates with this change. Several other reports have shown that lipids can act as nucleation sites for amyloid aggregation, and our results are consistent with reports that emphasize the importance of lipid charge and composition in dictating the aggregation routes [62,87]. These intermediates are considered to be crucial participants in amyloid toxicity, as observed in this study, and their formation may depend on lipid–protein electrostatic interactions. Furthermore, the effect of lipids on the aggregation kinetics observed in our ThT experiments supports previous research demonstrating that the presence of lipids can accelerate or alter the trajectory of protein aggregation. This shift in the aggregation pathway away from pure fibril formation to a combination of oligomers and fibrils has been noted in studies on several prion proteins, where lipid environments lead to differences in aggregation patterns [88,89]. These findings add to the growing body of research showing that lipids can act as modulators of amyloid fibrillation in various protein systems, including insulin, and contribute new insights into the structural and cytotoxic characteristics of lipid–associated insulin oligomers. Our TEM images, combined with DLS data, clearly showed the presence of both fibrils and oligomeric intermediates in insulin–DMPG mixtures, whereas insulin alone predominantly formed fibrils without discernible oligomers at later stages of aggregation. This observation is corroborated by the literature reports, where lipid–induced stabilization of small oligomeric species has been identified as a common phenomenon in amyloid systems [90]. This similarity suggests that the formation of lipid–bound oligomers may be a general mechanism by which lipids influence the amyloid aggregation of different proteins.

Furthermore, a shift in secondary structures was also observed for insulin while transitioning from monomeric to aggregated structures. The shift from α–helical structures in native insulin to β–sheet–rich structures during aggregation was confirmed by CD spectroscopy, consistent with other amyloid–forming proteins, where β–sheet formation is critical for fibrillation. Our echo studies showing that amyloidogenic proteins tend to adopt β–sheet–rich conformations when transitioning to aggregated states in lipid environments [91]. The FTIR data, showing β–turns in the oligomers (carbonyl stretch at 1660 cm^−1^), suggest that these intermediates are structurally distinct from both native and mature fibrils of insulin. These findings reinforce the idea that lipid–induced oligomers may have distinct folding patterns, which could contribute to their stability and toxicity. Insulin oligomers were also shown to be the most cytotoxic aggregate species in our study. This toxicity is believed to be correlated with the oligomer structure of amyloids, in which hydrophobic surfaces are exposed in β–sheets [53,92,93]. This hydrophobic quality can allow oligomers to infiltrate cell membranes and cause cell death. Our finding that insulin oligomers in the presence of DMPG lipids are more toxic to fibroblasts further supports the hypothesis that lipid–induced oligomers may share similar toxicity mechanisms across different amyloid proteins. To assess the cytotoxicity of DMPG–generated oligomers, insulin oligomers were stabilized by DMPG for up to 24 h and separated from high–molecular–weight fibrils by centrifugation. While the oligomers exhibited toxicity, the DMPG–insulin fibrils exhibited low toxicity, as shown in Figure 2n. In contrast, insulin aggregates formed in the absence of lipids contained negligible amounts of oligomers in the centrifuged supernatant, as confirmed by TEM images and CD data. The CD spectra displayed a signature corresponding to a distorted α–helix, indicative of unreacted or dissociated monomers, rather than the mixed parallel and antiparallel β–sheet structures characteristic of oligomers (Table S1). In this case, the remaining monomers completely converted into fibrils within 24 h (Figure 2f). These findings suggest that insulin oligomers formed without lipids are not sufficiently stable, and therefore, are less likely to contribute to oligomer–mediated toxicity, in contrast to the more persistent oligomers formed in the presence of DMPG lipids. The increased toxicity of lipid–associated insulin oligomers in NIH3T3 fibroblast cells observed in our study is consistent with a wealth of literature suggesting that oligomeric intermediates, rather than fibrils, are the primary toxic species in amyloid–related diseases. However, the specific lipid–driven mechanism underlying the cytotoxicity of these intermediates remains unclear, and it is uncertain which part of the insulin molecule (A or B chain) interacts with DMPG to promote toxicity. Although lipid–protein interactions are generally thought to influence cytotoxicity indirectly by modulating aggregate formation [94,95,96], future structural studies using NMR or cryo–EM could offer more detailed insights into these interactions.

The choice of anionic lipids, specifically DMPG, was based on their well–established ability to mimic negatively charged biological membranes, which are known to play a critical role in the aggregation of amyloidogenic proteins, including insulin. In addition, we also used zwitterionic DMPC lipids below their critical micelle concentration to study their effect on insulin aggregation (Figure S3). While nanomolar concentrations were sufficient for observing the effects of DMPC, we did not use DMPG in the nanomolar concentration range because electrostatic interactions require higher surface density. At nanomolar concentrations, the number of DMPG molecules is likely to be insufficient to establish the required electrostatic interaction with insulin. For these reasons, nanomolar concentrations of DMPG were not used. Instead, a sub–CMC micromolar range was chosen to balance biological relevance, reproducibility, and assay sensitivity.

These lipids serve as model systems to investigate membrane–assisted aggregation processes, which are highly relevant to understanding the molecular mechanisms underlying amyloid formation in both physiological and pathological contexts. While DMPG may not directly represent a specific environmental factor in human disease, it serves as an excellent model system to investigate the electrostatic interactions and lipid–protein dynamics that drive aggregation. These studies are crucial for understanding lipid–induced insulin aggregation and toxicity, particularly in scenarios such as its overproduction in pancreatic cells or its injection into the dermis and subcutaneous fat, where it can interact with various lipids. The significance of our work lies in advancing the broader understanding of how lipids influence amyloid aggregation by steering it through alternative pathways that govern the formation of intermediate species and their associated cytotoxicity. By investigating the specific effects of DMPG on insulin aggregation, we shed light on key mechanisms that could guide the development of therapeutic strategies aimed at targeting membrane interactions to reduce aggregation–related cytotoxic effects. Our study adds to the growing understanding of how lipid environments influence protein aggregation and toxicity [53,93,94,97,98,99]. The interaction of insulin with DMPG, leading to the formation of toxic oligomers, has broad implications for amyloid–related diseases, especially those where insulin aggregation is implicated. Moreover, this research could have therapeutic implications in designing inhibitors that target specific lipid–protein interactions to prevent toxic oligomer formation. However, it remains to be investigated whether these oligomers further seed insulin monomers to form structurally distinct fibrils of insulin. Furthermore, since insulin and its bioavailability in its native form are involved in diabetes, it is crucial to understand whether insulin oligomers affect cross–seeding of other amyloidogenic proteins [100]. Moreover, insulin’s ubiquitous presence in various organs also makes it imperative to understand its crosstalk with other amyloidogenic proteins such as IAPP, Aβ, and αSyn. Future studies should focus on identifying the specific molecular interactions between insulin and DMPG to uncover which regions of the insulin molecule are responsible for lipid–induced aggregation. Additionally, understanding how these lipid–associated oligomers exert their toxic effects on cells could pave the way for novel therapeutic strategies aimed at mitigating amyloid toxicity in both diabetes and other amyloid–associated conditions. In conclusion, our findings demonstrate that anionic lipids such as DMPG significantly modulate insulin aggregation, leading to the formation of small, toxic oligomers that differ structurally from both monomers and fibrils. These results are consistent with existing studies on other amyloid proteins and suggest that lipid–induced oligomers may represent a key target for understanding and treating amyloid diseases.

## 4. Conclusions

In summary, our study reveals that the presence of anionic lipids during insulin aggregation profoundly influences the mechanism of fibril formation. Notably, negatively charged lipids promote the formation of cytotoxic oligomeric species, which are absent when insulin undergoes homotypic interactions or associates with zwitterionic lipids such as DMPC. Furthermore, the interaction between insulin and anionic lipids drives the formation of more rigid fibrils compared with those formed in the presence of zwitterionic lipids. These findings highlight the pivotal role of lipid charge in shaping insulin’s structural and toxicological properties, offering valuable insights into the molecular mechanisms underlying amyloid diseases.

## Data Availability

All data is available at: https://osf.io/a4py6/?view_only=8ab908d92e854ba28853b534834119c2 (accessed on 4 July 2025).

## Supplementary Materials

The following supporting information can be downloaded at: https://www.mdpi.com/article/10.3390/biom15070994/s1, Hydrodynamic radius determination for insulin monomers at pH 3 using DLS data; ^1^H NMR spectra of insulin monomers in the presence of DMPG; ThT fluorescence kinetics and TEM of insulin with DMPC; TEM images (of total, pellet, and supernatant fractions insulin monomer reactions with DMPC); structure of insulin and DMPG and DMPC lipids; ^1^H NMR spectra of insulin aggregates generated with and without DMPG at 6 h or 24 h; natural–abundance ^13^C MAS NMR spectra of insulin aggregates prepared in the presence of DMPG or DMPC and without lipids; Bestsel structural analysis of CD spectra of insulin–DMPG and insulin aggregation [101].
