# Supplementary material for: Anionic Lipid Catalyzes the Generation of Cytotoxic Insulin Oligomers
Source: Biomolecules. 2025 Jul 11;15(7):994. doi: 10.3390/biom15070994 (PMC12292867; doi:10.3390/biom15070994)
Supplement: Supplementary file 1 [file biomolecules-15-00994-s001.zip › biomolecules-3698880-supplementary.pdf]

SUPPORTING INFORMATION

Anionic lipid catalyzes the generation of cytotoxic insulin oligomers

Jhinuk Saha[1,2,3*], Audrey Wolszczak[1,2], Navneet Kaur[1,2], Malitha C. Dickwella Widanage[1,2,3], Samuel D McCalpin[1,2,3], Riqiang Fu[1], Jamel Ali[1,2], Ayyalusamy Ramamoorthy[1,2,3*]

[1]National High Magnetic Field Laboratory, Florida State University, 1800 E. Paul Dirac Drive, Tallahassee, FL 32310, United States

[2]Department of Chemical and Biomedical Engineering, FAMU-FSU College of Engineering, Florida State University, 2525 Pottsdamer St., Tallahassee, FL 32310, United States

[3]Institute of Molecular Biophysics, Florida State University, 91 Chieftan Way, Tallahassee, FL 32304, United States

Authors for correspondence:

Jhinuk Saha (js23cp@fsu.edu) and Ayyalusamy Ramamoorthy (aramamoorthy@fsu.edu)

SUPPORTING INFORMATION

Supplementary figure 1

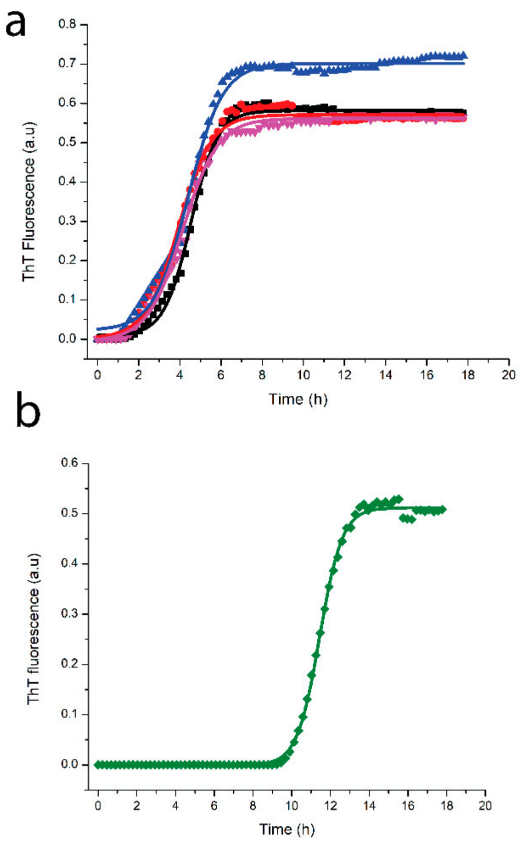

c

	2µM	4µM	6µM	8µM	Control insulin
Lag Time (h)	2.5	1	1	1	10

Figure S1. Sigmoidal fits of ThT fluorescence kinetics (a) of 80 µM insulin with 2 (black), *4* (red), *6*(blue), and *8 µM* (magenta) *DMPG (b) no lipids 0* (green) *and (c) Aggregation lag-times for each reaction estimated from extending a tangent line from point of nucleation (inflection point) back to the time axis.*

Supplementary figure 2

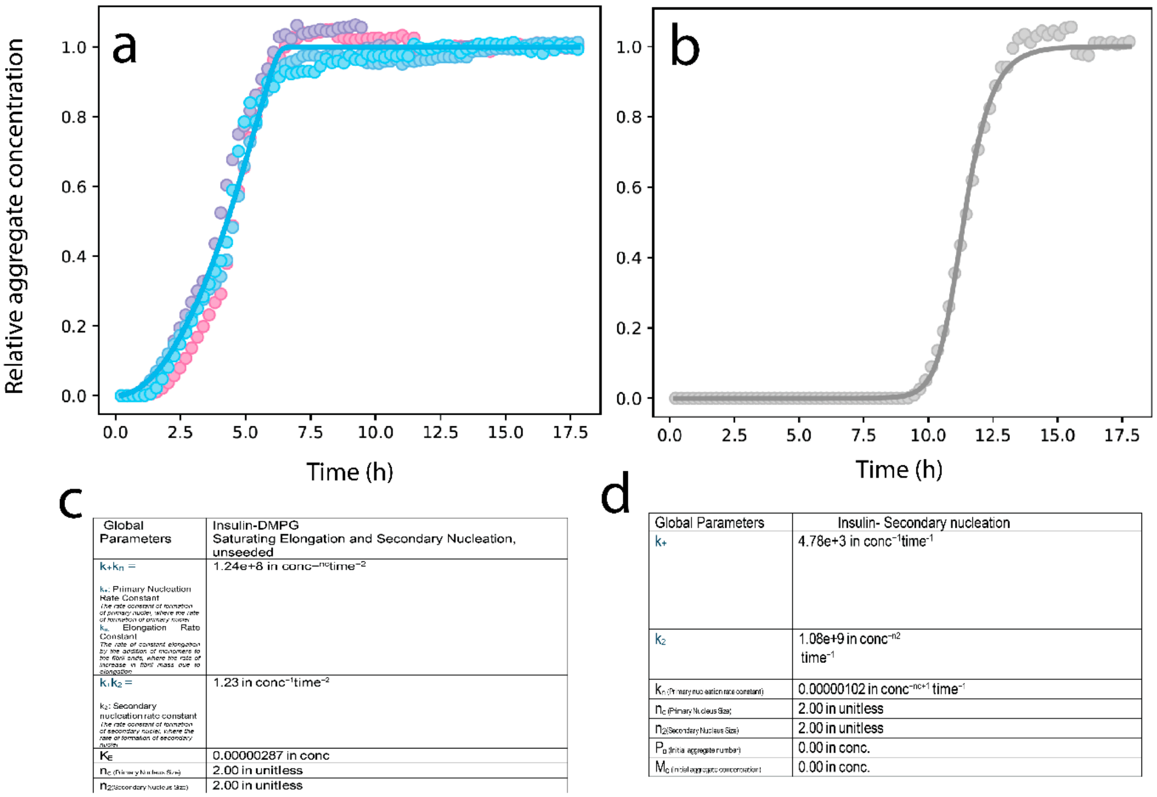

c

Global Parameters	Insulin-DMPG Saturating Elongation and Secondary Nucleation, unseeded
k_+k_n = k_+: Primary Nucleation Rate Constant *The rate constant of formation of primary nuclei, where the rate of formation of primary nuclei* k_n: Elongation Rate Constant *The rate of constant elongation by the addition of monomers to the fibril ends, where the rate of increase in fibril mass due to elongation*	1.24e+8 in conc^{-nc}time^{-2}
k_+k_2 = k_2: Secondary nucleation rate constant *The rate constant of formation of secondary nuclei, where the rate of formation of secondary nuclei*	1.23 in conc^{-1}time^{-2}
K_E	0.00000287 in conc
n_c (Primary Nucleus Size)	2.00 in unitless
n_2 (Secondary Nucleus Size)	2.00 in unitless

d

Global Parameters	Insulin- Secondary nucleation
k_+	4.78e+3 in conc^{-1}time^{-1}
k_2	1.08e+9 in conc^{-n2} time^{-1}
k_n (Primary nucleation rate constant)	0.00000102 in conc^{-nc+1} time^{-1}
n_c (Primary Nucleus Size)	2.00 in unitless
n_2 (Secondary Nucleus Size)	2.00 in unitless
P_0 (Initial aggregate number)	0.00 in conc.
M_0 (Initial aggregate concentration)	0.00 in conc.

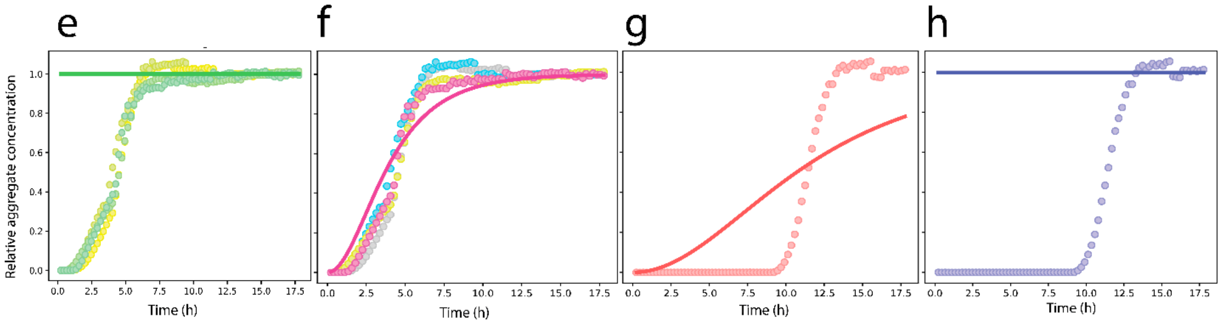

Figure S2. Global fits for (a) insulin aggregation with 2 (pink), 4 (purple), 6 (light blue), and 8 (dark blue) µM of DMPG . (b) Insulin aggregation in the absence of lipids using Amylofit software. Fitting type and parameters for panel (a) and (b) are given in (c) and (d) respectively. Global fits of insulin aggregate samples using the fragmentation model (e and h) and primary nucleation model (f -g).

Supplementary figure 3

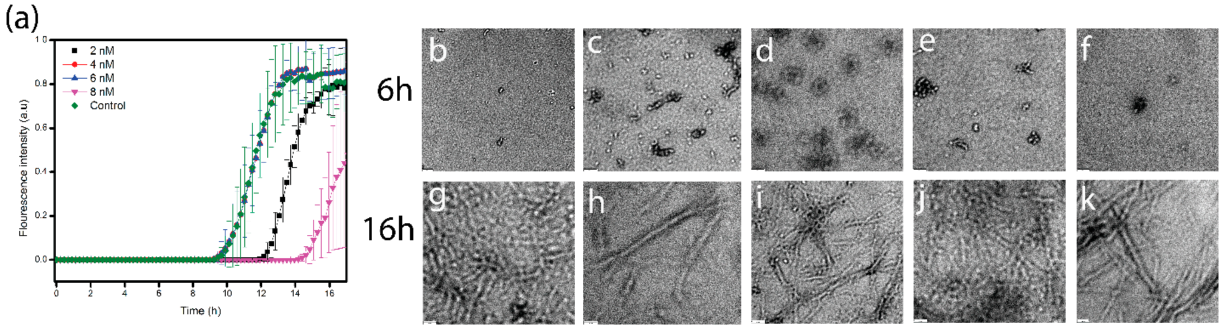

Figure S3. ThT fluorescence kinetics (a) of 80 µM insulin with 0 (◆), 2 (■), 4 (●), 6(▲), and 8 (▼) *nM (←) DMPC. Insulin\and DMPC were incubated in 10 mM sodium phosphate buffer (pH 3, 150 mM NaCl, 50 µM ThT) at 37 °C and with agitation at 700 rpm while insulin monomers were freshly prepared in 10 mM sodium phosphate buffer at pH 3. (b-k) TEM images of insulin aggregates generated in the ThT fluorescence assay with 2(b, g), 4(c, h), 6(d, i), 8 (e, j) nM DMPC and without DMPC (f, k); these aggregates were collected after 6h (c-g) and 16h (h-l). Scale bar is 200 nm.*

Supplementary figure 4

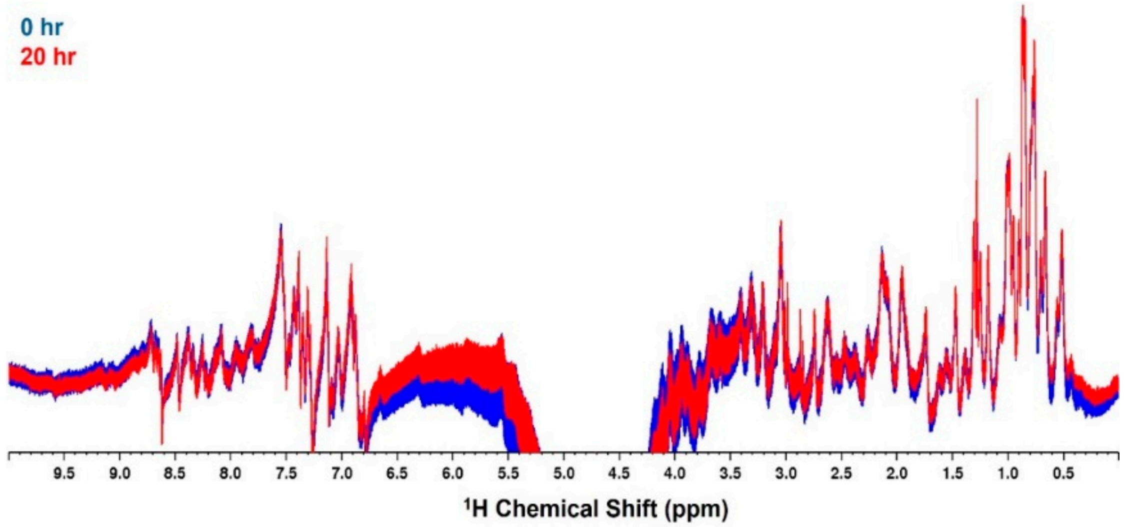

Figure S4. ^{1}H NMR spectra of insulin monomers in the presence of DMPG at 37 °C in 10 mM sodium phosphate pH 3 without any shaking from 0 to 24 h acquired on a 700 MHz NMR spectrometer using a cryoprobe.

Supplementary figure 5

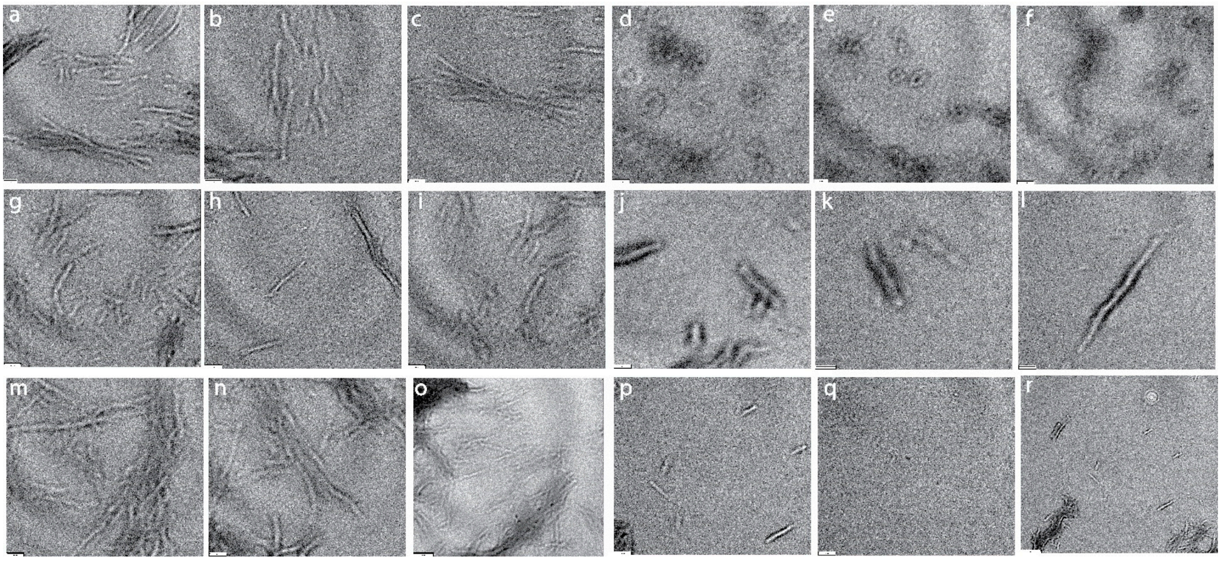

Fig. S5. Additional TEM images of insulin fibrils generated with: i)6 µM DMPG (a, b, and c are pellet fractions; d, e, and f are supernatant fractions); ii) 6 nM DMPC (g, h,and i are pellet fractions; j, k, and l are supernatant fractions); iii) no lipids (m, n, and o are pellet fractions; p, q, and r are supernatant fractions). Samples were run at 19000 x g for 30 min to separate pellet and supernatant after 24 h from the start of reactions. Scale bar is 100 nm (a-q) and 200 nm(r).

Supplementary figure6

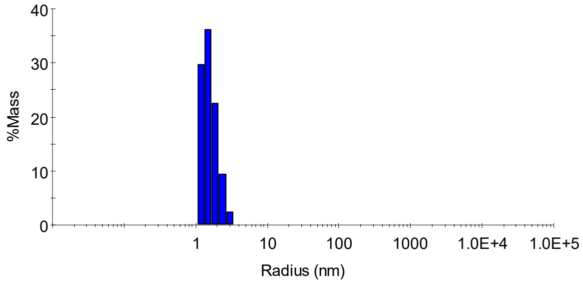

Figure S6. Dynamic light scattering (DLS) profile of freshly prepared insulin monomers (10 µM) in 10 mM phosphate buffer pH 3.

Supplementary figure 7

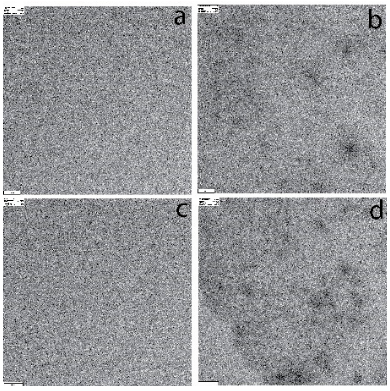

Figure S7. TEM images of 6 µM DMPG in 10 mM sodium phosphate buffer with 150 mM NaCl at pH 3.0 at 25,000 (a,b) and 15,000 (c,d) times magnifications for different areas of the TEM grid. The scale bar is 100 nm (a,b) and 200 nm (b,c).

Supplementary figure 8

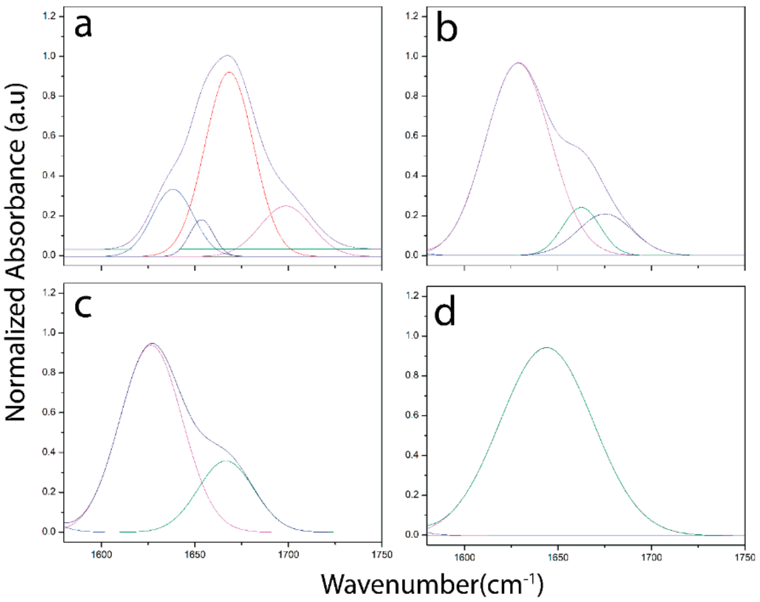

Figure S8. Peak deconvolution analysis of FTIR spectra obtained from: (a) insulin oligomers with DMPG, (b) insulin fibrils generated in presence of DMPG lipids, (c)insulin fibrils without lipids, and (d) insulin monomers.

Supplementary figure 9

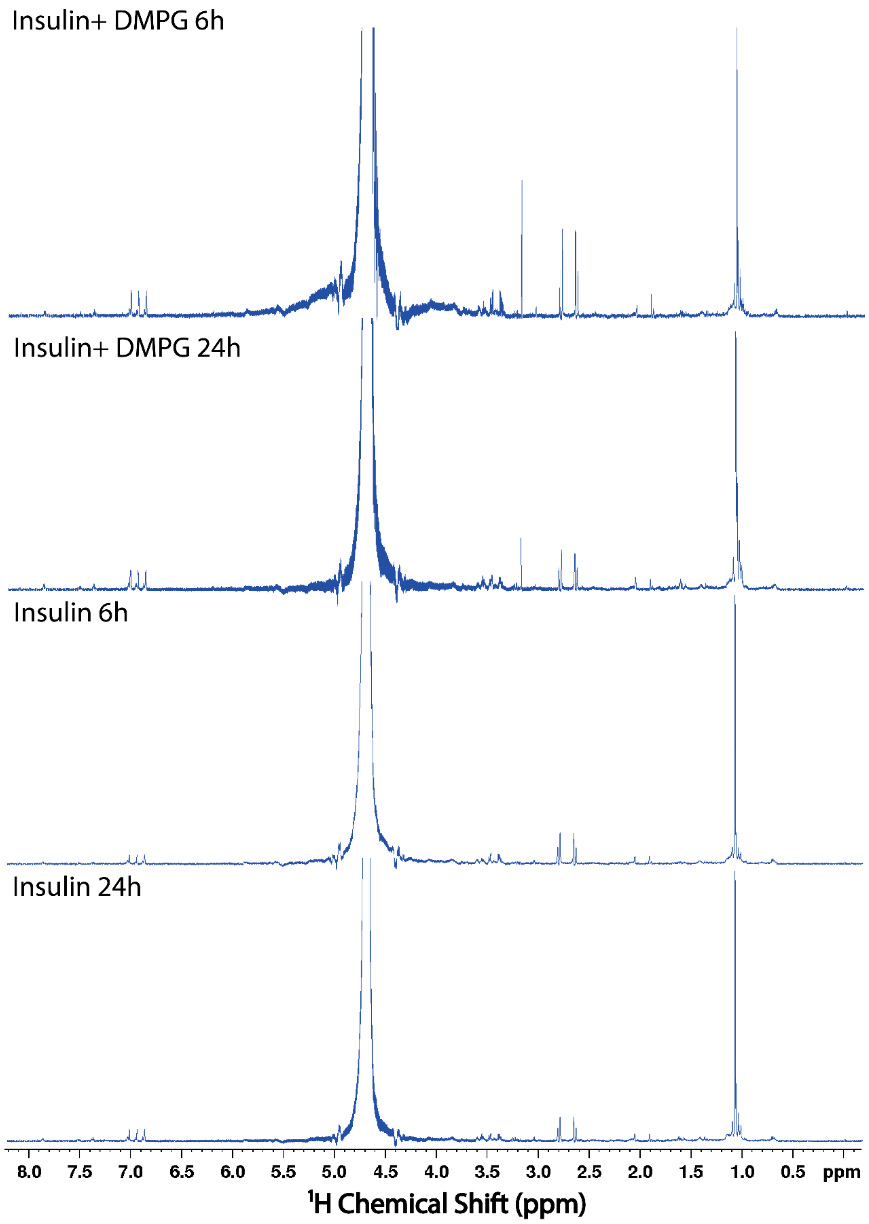

Figure S9. ¹H NMR spectra of insulin aggregates generated with and without DMPG at 6h or 24h upon shaking at 700 rpm at 37 °C in 10 mM sodium phosphate pH 3. Spectra were acquired on a 700 MHz NMR spectrometer using a cryoprobe.

Supplementary figure 10

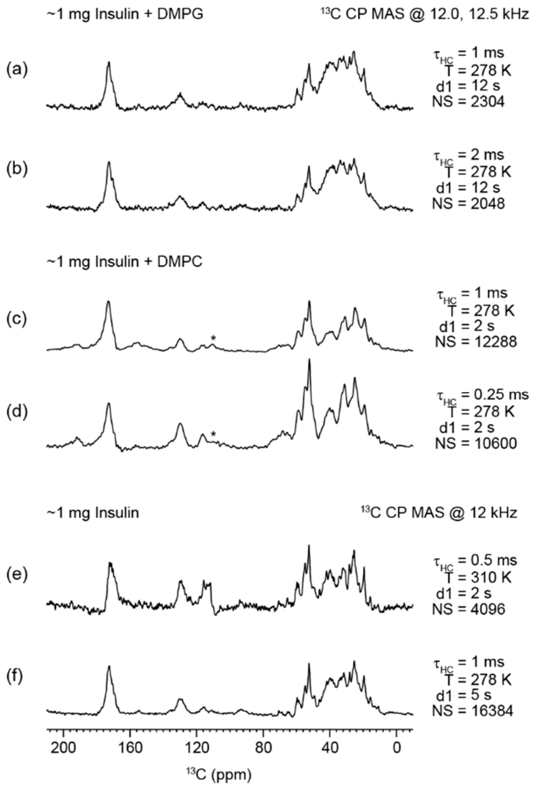

Figure S10. Natural-abundance ^{13}C MAS NMR spectra of insulin aggregates prepared in presence of DMPG or DMPC (a,b,d,e) and without lipids (c,f). Other NMR experimental parameters used to acquire these spectra are as mentioned in Figure 3.

Supplementary figure 11

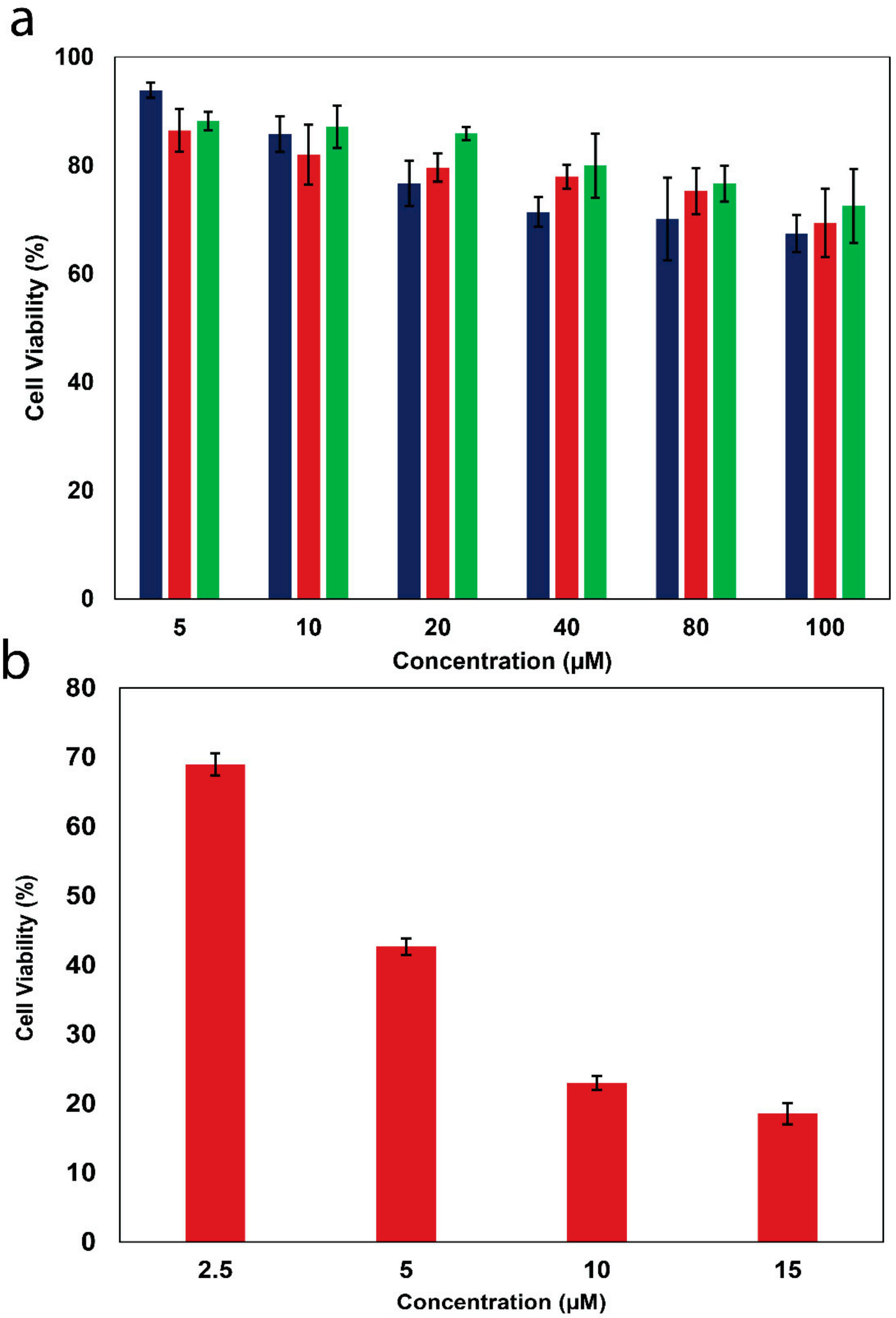

*Figure S11. CCK-8 cytotoxicity assay on NIH3T3 cells for: (a) 5, 10, 20, 40, 80 and 100 µM insulin fibrils generated with DMPG (*blue*), DMPC (*red*), and without lipids (*green*); (b) 2.5, 5, 10, and 15 µM insulin oligomers catalyzed by DMPG.* To maintain consistency with the ThT aggregation assay (Figure 1), we used the same lipid concentrations in the cytotoxicity experiments: 6 µM DMPG and 6 nM DMPC with 80 µM insulin. The samples were incubated at 70 °C under constant agitation (700 rpm) for 24 hours, followed by centrifugation at 17,000 rpm to separate the pellet (fibrillar fraction) and the supernatant (containing oligomers

and/or monomers). Each fraction was lyophilized and subsequently reconstituted to the desired protein concentrations to assess concentration-dependent cytotoxic effects.

Supplementary figure 12

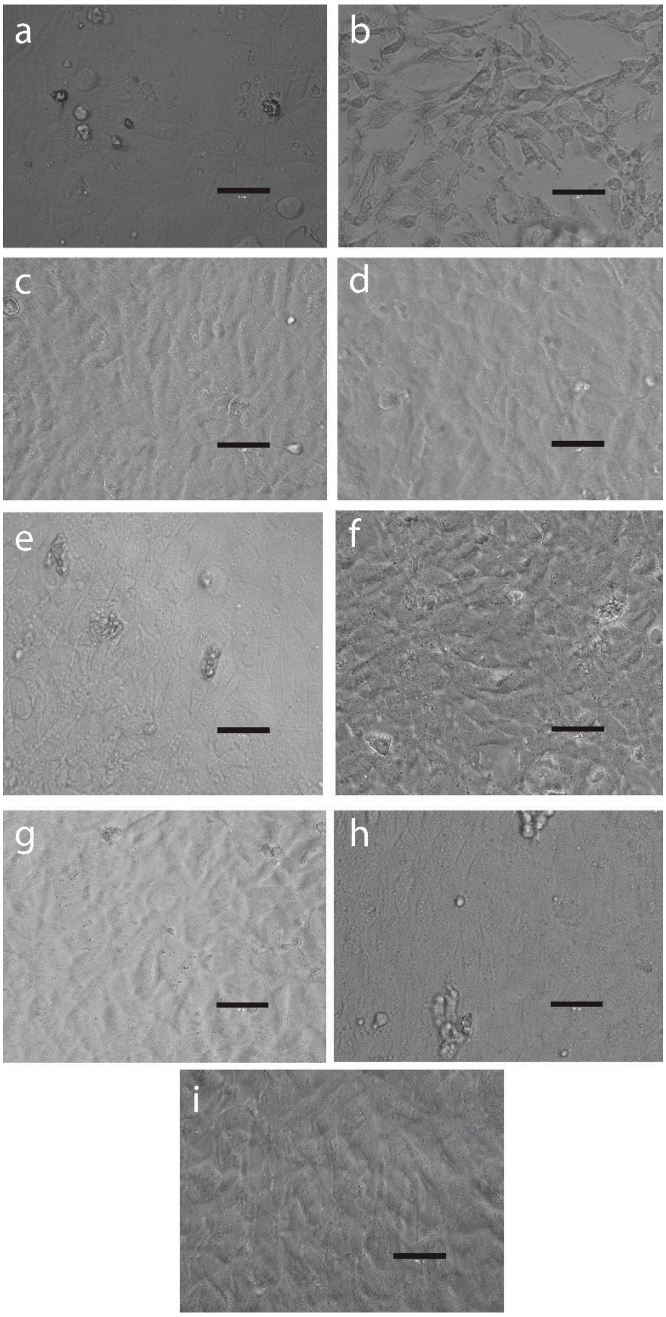

Figure S12. Bright-field microscopy images of 5 µM (a, c, e, g) and 10 µM (b, d, f, h) insulin species: oligomers generated in the presence of DMPG (a,b), insulin fibrils generated in the presence of DMPG (c,d), insulin fibrils generated in the presence of DMPC (e,f), insulin fibrils generated without any lipids (g,h) and control cells without fibrils (i). Scale bar is 50 µm.

Supplementary figure 13

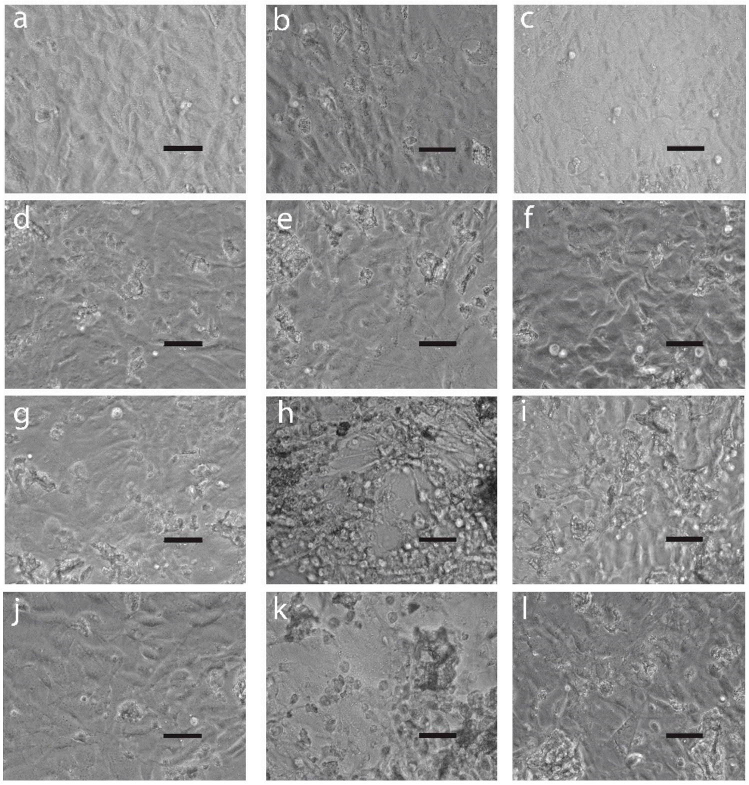

Figure S13. Bright-field microscopy images of 20, 40, 80 and 100 µM insulin fibrils generated in the presence of DMPG (a, d, g, and h, respectively), insulin fibrils generated in the presence DMPC(b, e h, and k, respectively), insulin fibrils generated without any lipids (c, f, i, and l, respectively). Scale bar is 50 µm.

Supplementary figure 14

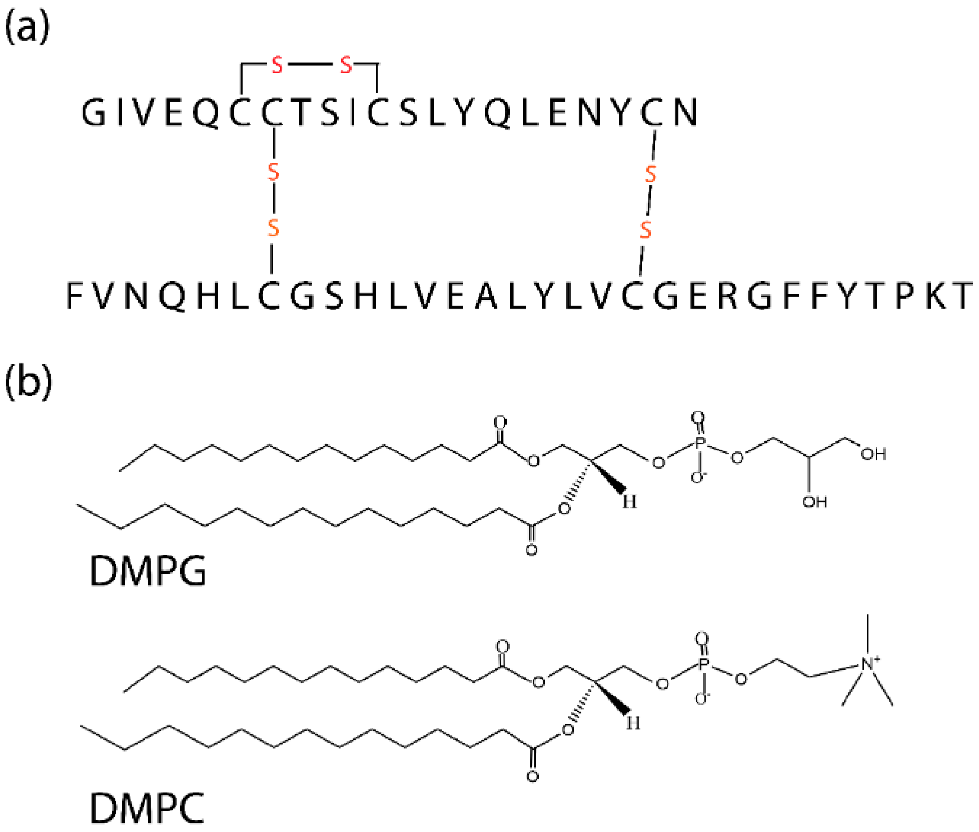

Figure S14. Molecular structures of insulin and DMPG and DMPC lipids used in this study.

Supplementary Table 1

SAMPLE NAME → / SECONDARY STR(%) ↓	Insulin Monomer	Insulin-DMPG Supernatant	Insulin control Supernatant	Insulin-DMPG Pellet	Insulin control Pellet
Helix1 (regular)	100	0	0	0	0
Helix2 (distorted)	0	0	100	50	50
Anti1 (left-twisted)	0	50	0	0	0
Anti2 (relaxed)	0	0	0	0	0
Anti3 (right-twisted)	0	0	0	0	0
Parallel	0	50	0	0	0
Turn	0	0	0	50	50
Others	0	0	0	0	0

Table S1. Bestsel structural analysis of CD spectra of insulin-DMPG and insulin aggregation reaction shown in Figure 2c and 2f in the main text. It should be noted that insulin monomers are helical but not 100% helical[1]; therefore, the estimated 100% helical structure is the direct prediction of Bestsel.

REFERENCES

1. Hua, Q.; Weiss, M.A. Comparative 2D NMR Studies of Human Insulin and Despentapeptide Insulin: Sequential Resonance Assignment and Implications for Protein Dynamics and Receptor Recognition. *Biochemistry* **1991**, *30*, 5505–5515, doi:10.1021/bi00236a025.
